# Parasites in Food Webs: Untangling the Entangled Bank

**DOI:** 10.1371/journal.pbio.1001580

**Published:** 2013-06-11

**Authors:** Jonathan Chase

**Affiliations:** Freelance Science Writer, Saint Louis, Missouri, United States of America

In the last paragraph of *On the Origin of Species*, Darwin described the interactions among birds, insects, and plants within an “entangled bank” as a metaphor to emphasize that although this might seem complex, these species and their interactions were formed by a set of basic laws (i.e., natural selection). Ecologists interested in the nature of webs of species interactions and their influence on biodiversity often use the entangled bank metaphor, but rather than distilling this complexity into a basic set of underlying laws as Darwin had attempted, the entangled bank is often used in ecology to describe the seemingly unbound complexity of nature wrought with idiosyncrasy. Nevertheless, a handful of brave ecologists are determined to find order amidst the morass of complexity, even though their critics might say they are tilting at windmills.

**Figure:**
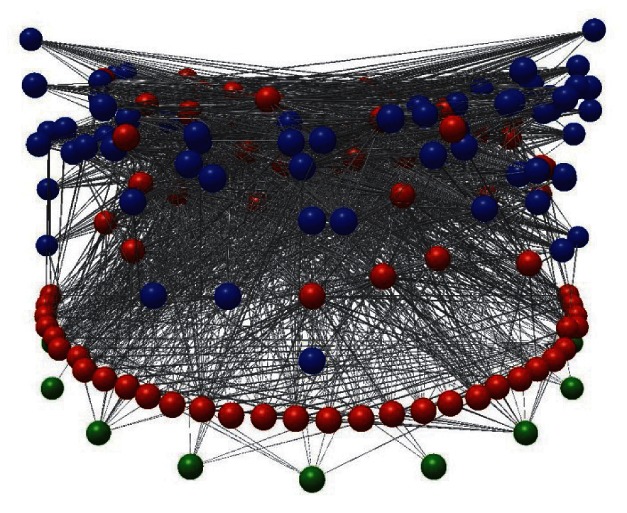
This image shows the network of 4,671 feeding interactions among 68 parasites (in blue) and 117 free-living taxa (green = basal taxa, red = consumers) in the food web of Estero de Punta Banda, Baja California, Mexico. **The vertical axis corresponds to trophic level.** Images produced by J.A. Dunne using Network3D software, available by request from jdunne@santafe.edu.

Armed with mathematical insight and a handful of empirically derived food webs, several ecologists in the 1970s and 1980s set out to determine whether regular structures in food web networks could provide a window into general ecological principles. A series of high profile publications led to the canonization of several such regularities, including constant ratios of predator to prey species, constant numbers of links per species, and limited levels of omnivory. Perhaps, they argued, these regularities were clues into scaffolding behind nature's complexity? There was only one problem: the regularities were untrue. The empirical food webs upon which they were based were…let's just say incomplete. They contained interactions among only a small fraction of the constituent species and were very poorly resolved; one of the analyzed networks, from a lake in Maine (United States), had ten species of fish at the top of the food web and two “species” of prey at the base—“phytoplankton” and “ooze.”

As the reality of the limitations of pattern analyses from poorly resolved food webs set in, many food web ecologists turned to detailed analyses of smaller subsets of species and their interactions. A small group, however, remained determined. They collected more detailed data of who eats whom, they developed more sophisticated computational tools to analyze these data, and they found that these more realistic food webs did indeed show consistent and predictable structure. For example, a graph theoretic “niche model,” with two inputs (the numbers of species and their connectance) and a one-dimensional ordering of species, where predators feed on contiguous subsets of the species, could reproduce most of the structural patterns observed.

Although these empirical food webs were an order of magnitude more complete than the earlier, “ooze-based” webs, the who eats whom links in their datasets largely ignored parasites. Perhaps because parasites are very diverse and participate in a large proportion of the links in a food web, recent studies have surmised this might fundamentally change how these networks are structured. Not to be deterred, in this issue of *PLOS Biology*, Jennifer Dunne and colleagues examined several food webs with highly resolved predator and parasite links to systematically evaluate the role of parasites in food web network properties.

Dunne and colleagues analyzed three versions of seven coastal marine and estuarine food webs. One version was “parasite naïve,” including only traditional predator-prey links, and another included the full complement of predator and parasite interactions; a third “intermediate” version of the food web included parasites, but not the extra complexity that emerges from concomitant links, where a predator eats its prey, but also incidentally eats the prey's parasites owing to their physical intimacy. Not surprisingly, including parasites increased the complexity of these networks (i.e., number of links per species and the overall connectance in the food web). Next, the researchers examined the numbers of links each species had to its prey (host) and predator (parasite) and found that the more complex datasets with parasites were more deviant from the expectations from a null distribution (maximum entropy) than the simpler parasite-less webs. Likewise, they predicted several network metrics (e.g., proportion of species among trophic levels, path length) from the above described “niche model” and compared those to observed values, finding less success predicting the observed structure of webs with parasites than without.

If Dunne and colleagues had stopped there, they might have made similar conclusions to previous studies: the addition of parasites fundamentally alters the structure of food webs. However, they knew that many metrics describing the structure of these food web networks are scale-dependent. Simply increasing the diversity of species and their links, as their analyses including parasites had done, is expected to change the structure of networks regardless of their specific feeding roles. This begs the question of whether parasites uniquely change food web structural properties relative to what would be expected from a generic increase in diversity and complexity. The answer, it turns out, was a small “yes” and a big “no.” Webs with parasites and concomitant links to them included had a higher frequency of intraguild predation (where a species that competes with another species also eats that species as a result of the concomitant links), and broader and more discontinuous feeding niches (e.g., many trematode parasites use snails, fish, and birds during different parts of their life cycles). However, when the full complement of parasitic interactions were included in the analysis, the greater deviations from the null and niche models, as well as changes in most of the metrics used to describe network structure, were what would have been predicted by increasing the diversity of any type of consumer or resource. Thus, most aspects of network structure changed with or without parasites as a scale-dependent response to increased diversity and complexity, not because there was anything particularly unique about there being parasites *per se* in these food webs.

In all, the results of Dunne and colleagues suggest that including a different class of species interactions (parasitism) does not fundamentally change most of the structural patterns of these interaction networks and that perhaps generalities might be forthcoming. At the same time, it is clear that the models that work reasonably well in less diverse systems (without parasites) are not adequate to describe the patterns that emerge when more realistically diverse communities are considered. The challenge now is to develop a theoretical construct that can adequately capture these apparent regularities that underlie the interconnections among life on Earth.

**Dunne JA, Lafferty KD, Dobson AP, Hechinger RF, Kuris AM, et al. (2013) Parasites Affect Food Web Structure Primarily through Increased Diversity and Complexity. doi:10.1371/journal.pbio.1001579**

